# Carotid artery longitudinal wall motion is associated with local blood velocity and left ventricular rotational, but not longitudinal, mechanics

**DOI:** 10.14814/phy2.12872

**Published:** 2016-07-20

**Authors:** Jason S. Au, David S. Ditor, Maureen J. MacDonald, Eric J. Stöhr

**Affiliations:** ^1^ Department of KinesiologyMcMaster UniversityHamiltonOntarioCanada; ^2^ Department of KinesiologyBrock UniversitySt. CatharinesOntarioCanada; ^3^ Discipline of Physiology & HealthCardiff School of SportCardiff Metropolitan UniversityCardiffWalesUK

**Keywords:** Carotid artery longitudinal wall motion, left ventricular rotation, ultrasound

## Abstract

Recent studies have identified a predictable movement pattern of the common carotid artery wall in the longitudinal direction. While there is evidence that the magnitude of this carotid artery longitudinal wall motion (CALM) is sensitive to cardiovascular health status, little is known about the determinants of CALM. The purpose of this integrative study was to evaluate the contribution of left ventricular (LV) cardiac motion and local blood velocity to CALM. Simultaneous ultrasound measurements of CALM, common carotid artery mean blood velocity (MBV), and left ventricular motion were performed in ten young, healthy individuals (6 males; 22 ± 1 years). Peak anterograde CALM occurred at a similar time as peak MBV (18.57 ± 3.98% vs. 18.53 ± 2.81% cardiac cycle; *t*‐test: *P *=* *0.94; ICC: 0.79, *P *<* *0.01). The timing of maximum retrograde CALM displacement was different, but related, to both peak apical (41.00 ± 7.81% vs. 35.33 ± 5.79% cardiac cycle; *t*‐test: *P *<* *0.01; ICC: 0.79, *P *<* *0.01) and basal rotation (41.80 ± 6.12% vs. 37.30 ± 5.66% cardiac cycle; *t*‐test: *P *<* *0.01; ICC: 0.74, *P *<* *0.01) with peak cardiac displacements preceding peak CALM displacements in both cases. The association between basal rotation and retrograde CALM was further supported by strong correlations between their peak magnitudes (*r* = −0.70, *P *=* *0.02), whereas the magnitude of septal longitudinal displacement was not associated with peak CALM (*r* = 0.11, *P *=* *0.77). These results suggest that the rotational mechanical movement of the LV base may be closely associated with longitudinal mechanics in the carotid artery. This finding may have important implications for interpreting the complex relationship between ventricular and vascular function.

## Introduction

Recent investigations have revealed a predictable and stable carotid artery longitudinal wall motion (CALM) pattern in healthy individuals, which retains both its pattern and magnitude over time (Ahlgren et al. 2012). Once thought to be breathing artifact, arterial motion in the longitudinal plane was first confirmed by cinematography of reflective beads sutured to the pig abdominal aorta (Tozzi et al. 2001), and has since been reported in the human common carotid artery (CCA) (Golemati et al. 2003; Persson et al. 2003; Soleimani et al. 2011). Due to the low motion velocities and thin arterial wall thickness, it has been difficult to accurately quantify CALM using standard techniques. With the use of more advanced 2D speckle tracking algorithms (O'Donnell et al. 1991, 1994; Larsson et al. 2011; Tat et al. 2015), longitudinal motion can be accurately quantified and has been shown to be of equal magnitude as radial expansion in both elastic (Cinthio et al. 2006; Tat et al. 2015) and muscular arteries (Cinthio and Ahlgren 2010).

Following initial descriptions of CALM, it is now apparent that the walls of the CCA move longitudinally, both in the direction of blood flow (anterograde) as well as in the direction opposing blood flow (retrograde), at different time points throughout the cardiac cycle (Golemati et al. 2003; Cinthio et al. 2006). Although the movement pattern may vary greatly between individuals, CALM has been demonstrated to remain stable within an individual over time (Ahlgren et al. 2012), making it a potential target for the noninvasive assessment of arterial properties in humans. The gold standard for the assessment of arterial stiffness, pulse wave velocity, has been extensively examined as a predictor for cardiovascular disease risk (The Reference Values for Arterial Stiffness’ Collaboration, 2010; Van Bortel et al. 2012; Townsend et al. 2015), and has been shown to correlate to the magnitude of CALM displacements (Taivainen et al. 2015; Yli‐Ollila et al. 2015). Preliminary cross‐sectional studies have reported differences in the magnitude of CALM in populations at high risk of developing cardiovascular disease, including older adults with diabetes, Indigenous Australians with periodontal disease and individuals with spinal cord injury (Zahnd et al. 2011, 2012; Tat et al. 2015). These studies indicate promise in CALM as a measurement of arterial health that is noninvasive, and feasible in a clinical setting.

Despite preliminary evidence of a link between CALM and cardiovascular health, the determinants of the phases of CALM remain unknown. Ahlgren et al. (2015) found no correlation between maximal longitudinal displacement of the porcine CCA intima‐media and wall shear rate. However, the authors did not separate CALM into anterograde and retrograde phases, and it may be that the forward shear stimulus can only explain the anterograde phase of motion. Likewise, the retrograde CALM phase is likely not determined by the local mechanical stimuli such as the frictional forces due to blood flow and transmural pressure forces, as anterograde shear stress would push the arterial wall in the forward direction. We have, therefore, approached this problem at an integrative systems level where we hypothesized that cardiac contraction would induce retrograde motion during early systole through a structural ventricular‐vascular coupling effect. Recent work by Zahnd et al. (2014) has demonstrated regional differences in CALM along the length of the CCA, quantifying an attenuation in movement with a coefficient of −2.5 ± 2.0% mm^−1^ distal to the heart. With a clear distal loss of motion magnitude along the CCA, it stands to reason that a cardiac factor may be affecting the magnitude of CALM. Yet, to date no studies have directly measured the influence of cardiac mechanics on CALM.

The purpose of this study was to evaluate the role of both local arterial and central cardiac mechanics on the timing and magnitude of specific CALM events in young, healthy individuals and thereby advance the understanding of CCA longitudinal mechanics. We hypothesized that the timing of retrograde CALM would be primarily linked to LV mechanics, and the timing of anterograde CALM would be linked to local blood flow events at the carotid artery.

## Methods

### Participants and ethical approval

Ten young healthy individuals (6 males, 4 females) were recruited for this study. All participants were between the ages of 18–35 years, and free of known cardiovascular disease. Informed consent in writing was obtained prior to participation in the study. The study protocol was submitted to, and approved by, the Hamilton Integrated Research Ethics Board and conforms to the *Declaration of Helsinki* concerning the use of human subjects as research participants.

### Experimental measures

All participants arrived at the lab between the hours of 0800–1000 in the fasted state, after refraining from exercise, alcohol and caffeine for >8 h. Participants then rested in the supine position for 10 min prior to any data collection. For data collection, participants were positioned in the left lateral decubitus position and equipped with three sets of single‐lead ECG to provide heart rate signals to two ultrasound units and a data collection system used to align the simultaneous measures during analysis.

#### Carotid arterial longitudinal motion

Longitudinal motion was assessed on the far wall of the right CCA, 2–5 cm proximal to the carotid bifurcation in the lateral plane using a 12 MHz linear‐array probe connected to a high‐resolution ultrasound machine (Vivid q, GE Medical Systems, Horten, Norway). A single focal point was positioned at the far wall and scanning depth was standardized at 2.5 cm to maintain a consistent sampling rate of 102.5 fps. Immediately prior to image acquisition, participants were asked to briefly hold their breath, as breathing artifact has been demonstrated to superimpose movement over longitudinal motion measures (Cinthio et al. 2005). Three to six heart cycles were recorded. Following acquisition, images were stored off‐line in the Digital Imaging and Communications in Medicine (DICOM) format, and separated into sequences of .jpg images for analysis. Carotid Arterial Longitudinal Motion (CALM) was measured using an in‐house speckle tracking algorithm (SpecTAT; MatLab, The MathWorks, Natick, MA) (Tat et al. 2015). In brief, the program uses a multiblock matching scheme to track two‐dimensional motion of the arterial wall. Gated to the R‐spike on the ECG, the lower edge of a reference kernel block (5.52 × 0.414 mm^2^) was manually placed over the media‐adventitia interface of the far wall. To improve resolution, a surrounding region of interest was interpolated to increase the number of pixels by a factor of four. For each successive frame, a normalized cross correlation function identified the peak correlation value for all possible kernel matches, and a shift in pixel position was taken to represent tissue displacement. A value above a lower boundary of 0.7 was considered acceptable for tracking (Farron et al. 2009). The coefficient of variation for day‐to‐day measurements of CALM magnitude in our laboratory (*n* = 10) was 7.8%, which indicates a good reproducibility between measurements, and compares well to other reports (Zahnd et al. 2011; Yli‐Ollila et al. 2013).

#### Echocardiography

In order to investigate contributions of LV contraction to events at the CCA, images were recorded from the parasternal and apical windows, simultaneously to CALM measurements. Echocardiographic image acquisition followed current guidelines for conventional variables (Lang et al. 2015) as well as for LV mechanics (Mor‐Avi et al. 2011). In order to standardize rotation analysis across individuals, the basal level was defined as the highest plane at which the mitral leaflets were visible. The apical level was defined as the imaging plane closest to the apex with no visible papillary muscles, as previously described (Stöhr et al. 2011). In addition, apical four‐chamber images were recorded. Images were taken with a 1.5–3.6 MHz phased‐array probe connected to a second ultrasound machine (Vivid q, GE Medical Systems) at >60 fps. Following acquisition, images were stored off‐line for further analysis using commercially available software (EchoPAC 110.0.2; GE Medical Systems, Horten, Norway). End‐diastolic volume, stroke volume, and posterior wall thickness were estimated from LV short‐axis images at the level of the base in M‐mode. 2D speckle tracking with drift compensation was used to quantify left ventricular basal and apical rotation and rotation velocity from LV short‐axis images, as well as basal septal displacement from the apical four‐chamber view. These traces were exported to a custom data processing software (2DStrainAnalysis Tool, Stuttgart, Germany) for further processing, with the purpose of achieving a relative time alignment of cardiac and CALM events (Burns et al. 2008) as described in more detail below.

#### Carotid blood velocity

For an assessment of timing of events, carotid mean blood velocity (MBV) was recorded simultaneous to vascular and cardiac imaging using a nonimaging 4 MHz pulsed wave probe at an insonation angle of 40° placed immediately proximal to the vascular probe on the CCA. This nonimaging probe was directly attached to an external spectral analysis system (model Neurovision 500 M TCD; Multigon Industries, Yonkers, NY) to determine intensity weighted mean blood velocity traces through a fast‐Fourier transform and these signals were subsequently sampled at 1000 Hz using commercially available hardware (PowerLab model ML 795; AD Instruments). To examine the relationship between the magnitude of carotid and cardiac events, MBV was also measured independent of other outcomes using a linear‐array probe in Duplex mode at 4 MHz (Vivid q, GE Medical Systems) at an insonation angle of 60° at 2–3 cm proximal to the carotid bifurcation, and processed as above. CCA shear rate was estimated using the equation, shear rate (1 sec^−1^) = (8*Blood Velocity at the CCA)/(CCA End Diastolic Lumen Diameter) (Parker et al. 2009). End diastolic diameters were analyzed offline using a custom‐designed semi‐automated edge tracking software (Artery Measurement System Image and Data Analysis, Tomas Gustavsson; Sweden) (Liang et al. 2000).

### Data analysis and definitions of CALM events

**Figure 1:**
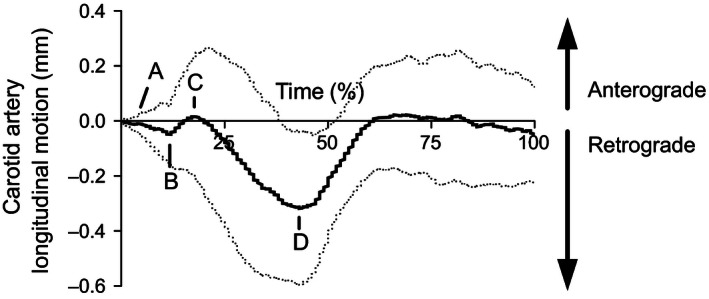
A typical CALM pattern expressed as percentage duration of a single cardiac cycle, gated to the R‐spike of the concurrently recorded ECG. Positive deflections indicate movement in the anterograde direction (i.e., in the direction of blood flow), whereas negative deflections indicate movement in the retrograde direction (i.e., in the direction of the left ventricle). Point A represents the onset of the first deflection in the retrograde direction. Point B represents the onset of the first anterograde movement. Point C represents the peak anterograde displacement. Point D represents the peak retrograde displacement. Dashed lines represent interpolated ±1 SD.

In order to account for different sampling rates and permit time alignment of parameters, all CALM and cardiac data were cubic‐spline interpolated to 600 points per cardiac cycle, with the R‐spike of the ECG denoting the first time point. Certain CALM events in the systolic period were consistently present in all individuals examined, as shown in Figure 1: (A) the onset of retrograde (negative) motion was determined as the first movement of the wall in retrograde direction; (B) the onset of anterograde (positive) motion was determined as the first movement in the anterograde direction, or the local positive peak of the 2nd derivative in the absence of a clear local minimum; (C) the peak anterograde (positive) displacement was determined as the maximal displacement in the anterograde direction away from the heart, or the local negative minimum of the 2nd derivative in the absence of a clear local maximum; and (D) the peak retrograde (negative) displacement was determined as the maximal displacement in the retrograde direction toward the heart.

Poor apical short‐axis image quality for one participant resulted in only that apical data being removed from analysis. For all statistical comparisons, the raw individual traces were used to prevent the loss of motion information with a filtering process. The total magnitude of CALM was determined as the difference between peak anterograde and peak retrograde displacement of the arterial wall, with more negative values indicating a greater retrograde movement during late systole. The CALM pattern was also divided into anterograde (point C–point B) and retrograde (point D–point C) phases, both reported in absolute terms. An average of 3–5 cardiac cycles were used to compare simultaneous data.

In addition to apical and basal rotation, we investigated longitudinal displacement of the LV to assess the relationship of CALM events to cardiac motion in the longitudinal plane. Owing to its anatomical proximity to the CCA, the basal septal segment of the 2D strain analysis package was isolated using the custom‐made 2DStrainAnalysis Tool (2DStrainAnalysis Tool, Stuttgart, Germany) and processed as above.

### Statistical analyses

Statistical analyses were performed on the Statistical Package for the Social Sciences (version 20.0.0 for Mac; SPSS, Chicago, IL). Data were checked for normality using the Shapiro–Wilk test. Dependent Student's *t*‐tests were used to assess differences in the timing between events at the same anatomical level (i.e., heart or carotid artery), expressed as a percentage of the cardiac cycle. For comparisons of local events at the CCA, where a time delay between events was not expected, nonsignificant *P*‐values from the *t*‐test and significant results from intraclass correlation coefficients (ICC) were interpreted as evidence for associations. Conversely, for comparisons between cardiac and CALM events, where a time delay between events was expected, only significant *P*‐values from the *t*‐test and significant results from intraclass correlation coefficients (ICC) were interpreted as evidence for associations. Because of the aforementioned expectation of a time delay between events at the heart and the carotid artery, relationships between the timing of CALM parameters with cardiac motion were determined from Consistency‐type ICCs with a two‐way random model.

To further explore associations revealed by the initial analyses, Pearson's correlations were applied to select variables that suggested associations between cardiac and CALM events. Analysis of data was performed on simultaneously collected heart cycles when possible, otherwise using an average of all available data for correlations. Statistical significance was set at *α *= 0.05.

## Results

**Figure 2:**
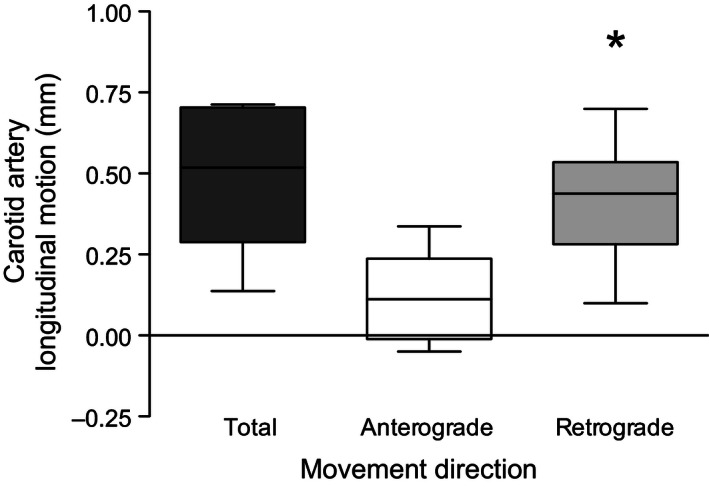
Total excursion, absolute anterograde, and absolute retrograde carotid artery longitudinal motion of the intima‐media layer of the common carotid artery wall. The box represents the 25th, 50th (median), and 75th percentiles. The bars represent the minimum and maximum values. *P < 0.01 compared with anterograde motion.

The participant characteristics (*n* = 10; 6 males, 4 females; 22 ± 1 years) are presented in Table 1. The maximal excursion of retrograde CALM was significantly larger than the maximal excursion of anterograde CALM (0.42 ± 0.18 vs. 0.13 ± 0.12 mm; *P *<* *0.01), and the total CALM displacement across one cardiac cycle was 0.50 ± 0.21 mm (Fig. 2).

**Table 1:** Participant characteristics (*n* = 10)

Variable	Mean ± SD
Sex (% Male)	60
Age (years)	22 ± 1
Height (m)	1.71 ± 0.08
Body mass (kg)	67.5 ± 9.9
SBP (mmHg)	116 ± 11
DBP (mmHg)	68 ± 6
MAP (mmHg)	87 ± 5
Heart rate (bpm)	61 ± 10
Cardiac parameters	
EDV (mL)	95 ± 15
Stroke volume (mL)	62 ± 10
PWT (mm)	0.88 ± 0.17
Peak apical rotation (°)	9.28 ± 5.30
Peak basal rotation (°)	−6.56 ± 3.03
Basal septal displacement (mm)	14.5 ± 2.0

SBP, systolic blood pressure; DBP, diastolic blood pressure; MAP, mean arterial pressure; CALM, carotid artery longitudinal motion; EDV, end‐diastolic volume; PWT, posterior wall thickness.

### Timing of CALM and cardiac events

**Figure 3:**
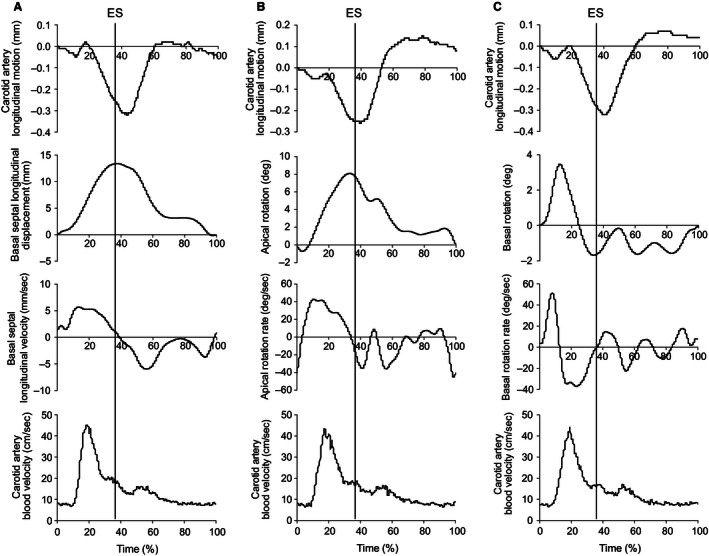
Group‐averaged traces for the simultaneous assessment of cardiovascular parameters aligning with the carotid artery longitudinal motion (CALM) pattern. Panel A: Alignment of CALM with basal septal displacement (positive deflections indicating movement towards the apex), basal septal velocity, and carotid blood velocity. Panel B: Alignment of CALM with apical rotation (positive deflections indicating counter‐clockwise rotation), rotation velocity and carotid blood velocity. Panel C: Alignment of CALM with basal rotation (positive deflections indicating counter‐clockwise rotation), rotation velocity and carotid blood velocity. Standard deviation lines have been removed for clarity. ES, end‐systole.

Figure 3 represents the group averaged interpolated traces for simultaneous collection of: (A) longitudinal displacement of the basal septum; (B) apical rotation; and (C) basal rotation. The timings of events are presented in Table 2. According to our statistical requirements for temporal associations, we identified that peak CALM displacements were related to both local (i.e., MBV) and upstream (i.e., LV) events. The time the vascular wall was at peak anterograde displacement (CALM point C) was not different from the time of peak blood velocity (*t*‐test *P *=* *0.94), with the MBV wave consistently reaching peak velocity before peak anterograde displacement of the wall (ICC: 0.79, *P *<* *0.01). The time at peak retrograde displacement (CALM point D) was different from the time of both peak apical (*t*‐test *P *<* *0.01) and basal (*t*‐test *P *<* *0.01) rotation, with peak cardiac displacements preceding peak retrograde wall displacements in both cases (apical ICC: 0.79, *P *<* *0.01; basal ICC: 0.74, *P *<* *0.01). This relationship was not found for peak basal septal displacement (*t*‐test *P *=* *0.09; ICC: 0.63, *P *=* *0.02). Although we also investigated the relationship between early CALM events (points A and B) with local blood velocity and LV motion, these associations did not meet both a priori statistical criteria and were therefore not evaluated further.

**Table 2:** Timing of events at the level of the left ventricle and the common carotid artery

Event pairing	Timing of events (% of heart cycle)			Mean difference 95% CI	*P*‐value	ICC [95% CI]	*P*‐value
	CALM point		Comparison event				
CALM point A with							
Onset of septal movement	3.30 ± 2.31	vs.	5.10 ± 2.47	−4.58 to 0.98	0.18	−0.32 [−0.77 to −0.35]	0.83
Apical prerotation peak	4.57 ± 1.99	vs.	1.00 ± 5.16	−7.08 to 2.22	0.25	0.17 [−0.61 to 0.78]	0.34
Basal prerotation peak	2.70 ± 3.57	vs.	12.90 ± 3.57	−14.13 to −6.27	<0.01	−0.50 [−0.85 to 0.15]	0.94
CALM point B with							
Arrival of blood velocity wave	9.83 ± 3.03	vs.	10.73 ± 1.41	−2.19 to 0.39	0.17	−0.07 [−0.42 to 0.29]	0.65
Peak basal prerotation velocity	9.50 ± 3.31	vs.	7.60 ± 1.78	−0.83 to 4.63	0.15	−0.03 [−0.62 to 0.58]	0.54
CALM point C with							
Peak blood velocity	**18.57 ± 3.98**	**vs.**	**18.53 ± 2.81**	−**0.80 to 0.86**	**0.94**	**0.79 [0.61 to 0.90]**	**<0.01**
Peak basal rotation velocity	18.30 ± 4.85	vs.	20.30 ± 4.69	−5.66 to 1.66	0.25	0.43 [−0.24 to 0.82]	0.10
CALM point D with							
Peak septal displacement	42.60 ± 6.42	vs.	39.40 ± 5.72	−0.55 to 6.95	0.09	0.63 [0.04 to 0.89]	0.02
Peak apical rotation	**41.00 ± 7.81**	**vs.**	**35.33 ± 5.79**	**2.23 to 9.10**	**<0.01**	**0.79 [0.31 to 0.95]**	**<0.01**
Peak basal rotation	**41.80 ± 6.12**	**vs.**	**37.30 ± 5.66**	**1.44 to 7.56**	**<0.01**	**0.74 [0.24 to 0.93]**	**<0.01**

CALM, Carotid artery longitudinal motion; point A, onset of first deflection in the retrograde direction; point B, onset of first anterograde movement; point C, peak anterograde displacement; point D, peak retrograde displacement; 95% CI, 95% confidence interval. Bolded events are interpreted as occurring at a similar time. Values are means ± SD.

CALM points differ slightly in timing as analysis of data was performed on simultaneously collected heart cycles when possible.

### Magnitude of cardiac and CALM events

**Figure 4:**
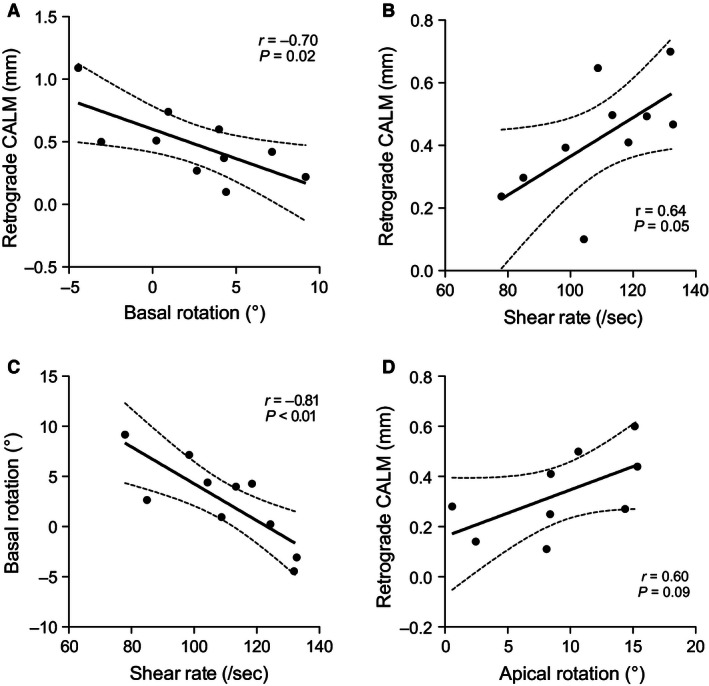
Correlations among the magnitude of events plotted with the 95% confidence intervals. (A) Basal rotation and retrograde CALM. (B) Carotid shear rate and retrograde CALM. (C) Carotid shear rate and basal rotation. (D) Apical rotation and retrograde CALM. Retrograde CALM and basal rotation are expressed as positive values.

Those variables that appeared to be temporally associated were further examined for associations between their magnitudes in order to gain greater insight into the strongest correlates between cardiac and CALM mechanics (Table 3 and Fig. 4). There was a moderate inverse correlation between retrograde CALM and participant height (*r* = −0.48; *P *<* *0.01), with taller individuals exhibiting less motion. There was no correlation between the magnitudes of anterograde CALM and carotid shear rate (*r* = −0.24; *P *=* *0.20). Retrograde CALM had a strong correlation with peak basal rotation (*r* = −0.70; *P* = 0.02) and shear rate (*r* = 0.63; *P *=* *0.05), but no statistical correlation with basal septal longitudinal movement (*r* = 0.11; *P *=* *0.77) or apical rotation (*r* = 0.63; *P *=* *0.09).

**Table 3:** Correlations between CALM displacements with cardiac and blood flow stimuli

event pairing	Pearson correlation coefficient	*P*‐value
Anterograde CALM with		
Carotid shear rate	0.08	0.83
Retrograde CALM with		
Height	−0.48	<0.01
Carotid shear rate	0.63	0.05
Basal septal movement	0.11	0.77
Basal septal velocity	0.27	0.46
Apical rotation	0.60	0.09
Apical rotation velocity	0.58	0.12
Basal rotation	−0.70	0.02
Basal rotation velocity	0.51	0.13

CALM, Carotid artery longitudinal motion; Retrograde CALM and basal rotation are expressed as positive values.

## Discussion

In this study, we show that longitudinal motion of the CCA (CALM) is associated with both local blood velocity and rotation of the LV base, but not with longitudinal septal displacement, thereby suggesting a physiological basis for the different phases of CALM previously observed. We propose that a complex relationship exists between the influences of local blood velocity and left ventricular rotation within the context of a structural ventricular‐vascular coupling theory, as discussed in detail below.

### Coupling theory

The role of cardiac contraction in CALM has been previously theorized in the literature but has never been reported (Cinthio et al. 2006; Ahlgren et al. 2012, 2015; Zahnd et al. 2012, 2014). In this study, we provide preliminary evidence for the influence of systolic cardiac events on the distinct longitudinal displacement of the arterial wall in the retrograde direction. Though the endocardium is not structurally continuous with the vascular wall of the CCA, the ventricular and vascular systems are anatomically directly linked by the aortic valve at the basal level of the LV, and indirectly by the ejection of blood from the LV. The present data suggest a previously unknown functional interdependence between the LV and CCA retrograde movement. These data are supported by Zahnd et al. (2014), who have demonstrated reductions in arterial wall motion along the length of the CCA, likely due to the distance from LV during systolic contraction. We, and others (Yli‐Ollila et al. 2015), also report that taller individuals exhibit less longitudinal motion of the CCA intima‐media complex, which is consistent with a reduction in CALM distal to the LV. Though this study shows a clear interaction between LV rotation and CALM, the exact determinants of interindividual variations in the CALM pattern and the potential use of CALM analysis for indications of vascular health remain to be determined.

### Timing of CALM events

Similar to previous reports, many CALM events were observed to be consistent across individuals (Golemati et al. 2003; Cinthio et al. 2006). Cardiac motion during systole appears to be related to motion of the retrograde segments of CALM, while the peak of the forward blood velocity wave is temporally linked with the peak anterograde segment of CALM. We found that the onset of retrograde CALM (point A in Fig. 1) occurred in concert with the onset of both basal septal longitudinal displacement and apical rotation, which is followed by a rapid increase in CCA blood velocity that coincides with the onset of anterograde wall motion (point B). However, we must note that these events did not meet both statistical requirements for association and we can only theorize their importance to the CALM pattern. As the blood velocity wave reaches peak magnitude, peak anterograde CALM displacement occurs (point C), and then retrograde motion resumes. We believe that during this period, the anterograde wall movement represents the interruption of the retrograde cardiac influence by the local mechanical blood velocity force (Nichols and O'Rourke 2005). When the anterograde blood velocity influence is waning, both LV longitudinal and rotational velocities reach peak instantaneous velocity. The now unopposed retrograde forces allow the arterial wall to reach peak retrograde displacement (point D) at end‐systole. At this point, there were strong associations between the timing of peak retrograde displacement with peak apical (ICC: 0.79), and basal rotation (ICC: 0.74), where cardiac motion preceded motion at the CCA. As both of these left ventricular motion parameters inevitably peak at end‐systole, we were unable to specify the greatest determinant of peak retrograde CALM displacement.

**Figure 5:**
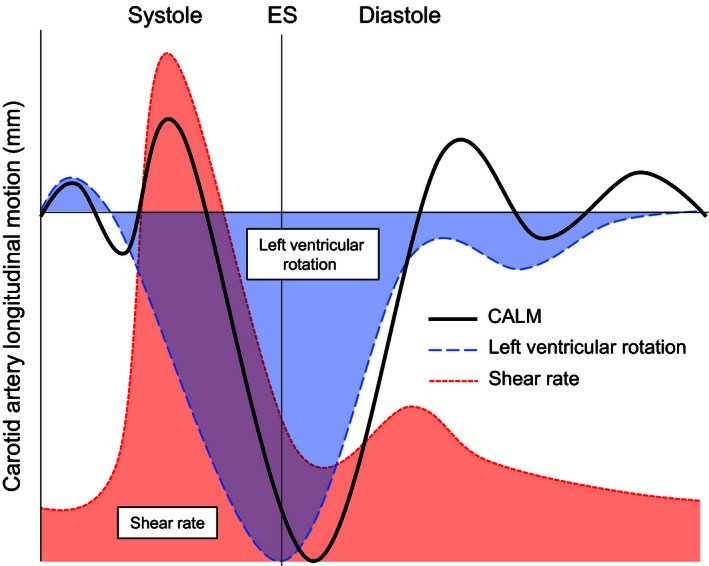
Schematicized theory of the influence of shear rate and left ventricular basal rotation on the CALM pattern as a summated function. Left ventricular rotation (blue) is theorized to begin at the first retrograde CALM displacement, which is briefly interrupted during early‐systole by shear rate (red) at the carotid artery. ES, end systole.

In summary, we hypothesize that motion at the LV initiates the movement pattern of CALM, which is interrupted during early‐systole by the arrival of the forward blood velocity wave. Thus, the systolic CALM pattern may be conceived as two separate functions (i.e., a sustained retrograde cardiac‐related wave that is briefly opposed by an anterograde blood velocity‐related wave and a subsequent return to retrograde motion) that superimpose upon each other to create the observed summated function (Fig. 5). We hypothesize that interindividual variations in the CALM pattern during this time represent the individual force summation properties, with a larger cardiac stimulus reflecting a more retrograde summated function (see the −1 SD trace in Fig. 1), and a larger blood velocity stimulus reflecting a more anterograde function (see the +1 SD trace in Fig. 1). In this model, the timing of systolic phases remains consistent across individuals, albeit with much different motion magnitudes. Increased stiffness of the CCA is likely to influence this relationship, but the impact of arteriosclerosis on CALM is currently unknown.

### Magnitude of CALM events

While our observations on the association between the timing of events yields insight into the factors associated with the pattern of CALM events during the systolic period, assessing the associations between the magnitude of events may offer further insight. In this regard, we observed no association between the magnitudes of the anterograde CALM component and the local shear rate stimulus, similar to previous reports in a well‐controlled model of the porcine carotid artery (Ahlgren et al. 2015). This lack of direct relationship may be due to the simultaneous competing influence of left ventricular rotation on the magnitude of movement of the carotid wall, as increases in both carotid shear rate and apical rotation occurred during the same period (see Fig. 5). Given that left ventricular contraction (whether displacement or rotation) approaches peak velocity during the same phase as peak carotid MBV (Fig. 3), it is not possible to isolate the contribution of the forward blood velocity wave to CALM event magnitudes from the confounding influence of ventricular motion in vivo. This has probably contributed to the lack of significant relationships between wall shear stress and CALM assessed by Ahlgren et al. (2015) through alpha‐ and beta‐adrenergic stimulation, as adrenergic modulation would also act on left ventricular systolic function, impacting the early systolic influence of the heart on the CALM pattern.

With respect to retrograde motion of the arterial wall, we hypothesized that movement of the basal septum would be the best candidate to predict the magnitude of retrograde CALM, as a pulling force would be applied to the vasculature directly through the aortic value in the same plane as the motion of the proximal aorta, and by extension the CCA. Contrary to our hypothesis, we did not observe any statistical relationship between the magnitudes of basal septal displacement and retrograde CALM. Instead, indices of LV rotation demonstrated relationships with the CALM pattern, suggesting that the natural twisting motion of the LV may have a stronger direct or indirect (through hemodynamics) effect on CALM than longitudinal LV displacement.

The consistency in which peak basal and apical rotation precede peak retrograde CALM displacement supports a direct relationship between LV rotation and arterial wall motion. Although the relationship between the LV base and CALM is supported by previous work suggesting that the kinetic energy of LV contraction appears to be greater at the LV base than the LV apex (Stöhr et al. 2014), the inverse linearity of the relationship between the magnitude of basal rotation and retrograde CALM seems to conflict with our coupling hypothesis. Instead of direct coupling, the LV base‐CALM association may be indirectly mediated by carotid shear rate, which was also associated with both basal rotation and retrograde CALM (Fig. 4). For example, it is possible that a greater basal rotation may increase the hemodynamic vortices observed in the basal aspect of the LV, leading to a greater turbulence of flow than at lower basal rotation (Kilner et al. 2000). This in turn may lead to an altered angle of flow, which has been shown to impact on endothelial cell sensing (Wang et al. 2013). Purposeful examination of these concepts presents an exciting opportunity for future research.

Fully elucidating the competing roles of LV rotation and the local impact of shear rate is a difficult task as these elements appear to act in a coordinated system, impacting both each other, as well as the CALM pattern. Although we were not able to determine a causal relationship from our resting data, we believe our observational data has set a framework for the investigation into the roles of these competing stimuli to predict the magnitude of CALM displacements. Additional stimulus‐response studies are required to further determine the factors that account of the magnitude of CALM events.

### Perspectives: measurement of arterial stiffness

Recently, CALM has been investigated as a novel indicator of arterial health (Svedlund and Gan 2011; Zahnd et al. 2011, 2012; Taivainen et al. 2015; Tat et al. 2015). While these studies have identified clear differences in the CALM pattern between healthy and clinical populations, our findings indicate that caution must be used when interpreting these differences. While the majority of studies have reported peak displacements within the CALM pattern, the absolute magnitude of CALM may not be comparable between different individuals or groups without considering the influence from the heart. Indeed, our results indicate that other systemic factors such as afterload, contractility and heart rate have the potential to indirectly influence CALM via their effect on upstream LV rotation (Gibbons Kroeker et al. 1995; Weiner et al. 2012). Indices that are largely magnitude‐independent such as the intramural shear strain index may be more appropriate indicators of arterial properties and have indeed shown sensitivity to detect differences in clinical populations (Cinthio et al. 2006; Tat et al. 2015). Zahnd et al. (2014) more recently theorized the use of an attenuation coefficient of motion along the CCA to enable tissue stiffness quantification, which may yield promising insights into a measure of arterial stiffness within the longitudinal plane (Zahnd et al. 2014). Finally, given the complex interaction between CALM, carotid shear rate and left ventricular rotation during systole, it may be of interest to examine ‘passive’ CALM events during diastole, as diastolic CALM would be largely unaffected from the influence of left ventricular rotation during the period when the aortic valve is closed. As new indices of arterial properties focused on the CALM pattern emerge, it is of utmost importance to consider the confounding contribution of cardiac motion when interpreting differences between healthy and clinical populations.

### Limitations

While the simultaneous measurement of cardiac function and CALM controlled for the natural beat‐to‐beat variation in heart cycles, we were limited in the measurements that could be obtained concurrently. Other indices of left ventricular contraction were not investigated, such as longitudinal strain, circumferential strain, or tissue Doppler velocity of the mitral annulus. Our decision to focus on rotation as well as basal septal displacement was based on our objectives for examining the origin of retrograde CALM with respect to regional motion of the left ventricle. It was not possible to measure arterial Doppler blood velocity and CALM at the same segment of the CCA, although velocity measurements were taken immediately proximal to the high‐resolution probe and should therefore not have caused a significant limitation to the present analyses. An in‐house speckle tracking system was used to measure CALM. While this particular program has not been externally validated, there is currently no criterion standard for vascular speckle tracking. Even so, CALM, as measured by our in‐house program, showed good variability (CV = 7.8%) compared with other groups (Yli‐Ollila et al. 2013). Finally, though this study may be underpowered in some specific comparisons, we were able to demonstrate strong correlations between outcome measures. The dual approach of examining associations between the timings as well as the magnitudes of cardiac and CALM events significantly increased our confidence in the determinants of CALM.

## Conclusion

In summary, we observed a temporal relationship between the blood velocity wave and anterograde CALM, while retrograde CALM was associated in both timing and magnitude with left ventricular rotation, but not longitudinal septal displacement, as originally hypothesized. This relationship between carotid artery movement and left ventricular rotation may have important implications for the regulation of local vascular stiffness properties, and the complex interaction between left ventricular and vascular function.

## Conflict of Interest

None declared.
