# Corrigendum

**DOI:** 10.14814/phy2.13036

**Published:** 2016-11-14

In Au et al. (2016), the following error was published in the Results section, Table 1.:

The unit of measurement used for PWT in Table 1., (mm), is incorrect and should instead be (cm).

The authors apologise for the error.
